# Bone microarchitectural degradation in hypertensive patients: a population-based study

**DOI:** 10.1007/s11657-026-01703-y

**Published:** 2026-04-27

**Authors:** Fabio Bioletto, Martina Bollati, Marco Barale, Chiara Lopez, Alessia Pusterla, Emanuela Arvat, Ezio Ghigo, Mauro Maccario, Massimo Procopio, Mirko Parasiliti-Caprino

**Affiliations:** 1 Division of Endocrinology, Diabetes and Metabolism, Department of Medical Sciences, University of Turin, Corso Dogliotti 14, 10126 Turin, Italy; 2 Division of Oncological Endocrinology, Department of Medical Sciences, University of Turin, Turin, Italy

**Keywords:** Hypertension, Anti-hypertensive medications, Trabecular bone score, Bone microarchitecture, Bone quality

## Abstract

***Summary*:**

Hypertension is associated with increased fracture risk. However, evidence on its association with BMD is conflicting. Here, we demonstrate that hypertensive patients have lower trabecular bone scores compared to normotensive subjects, despite similar BMD. This degradation of bone microarchitecture may help explain the increased skeletal fragility observed in hypertensive patients.

**Purpose:**

Hypertension is associated with an increased fracture risk. However, evidence on its association with bone mineral density (BMD) is conflicting, and data on bone microarchitectural quality are scarce. The in vivo effects of anti-hypertensive medications on bone quality are poorly explored. The primary aim of this study was to evaluate whether bone microarchitecture, non-invasively assessed by trabecular bone score (TBS), is altered in hypertensive patients. The association between anti-hypertensive medications and TBS was also evaluated as a secondary endpoint.

**Methods:**

We extracted individual data of 7053 subjects included in the 2005–2008 cycles of the National Health and Nutrition Examination Survey (NHANES), in which lumbar spine dual-energy X-ray absorptiometry (DXA) scans were acquired. TBS values were calculated from DXA images using dedicated software. The association between hypertension, anti-hypertensive medications, and bone outcomes was assessed by regression analyses, adjusted for relevant confounders.

**Results:**

Hypertension was independently associated with lower TBS values (*β* = −0.010; 95%CI, [−0.016, −0.003]; *p* = 0.008); on the contrary, no association was observed between hypertension and BMD at any site (lumbar spine: *p* = 0.362; total hip: *p* = 0.481; femoral neck: *p* = 0.298). No class of anti-hypertensive medications was significantly associated with TBS. Thiazide diuretics and angiotensin receptor blockers were associated with higher BMD values, whereas loop diuretics and non-dihydropyridine calcium channel blockers were associated with lower BMD values.

**Conclusions:**

Hypertension is associated with degraded bone microarchitecture, while no association is observed with bone mass. No significant relationships between anti-hypertensive medications and TBS were found. Associations between anti-hypertensive medications and BMD were consistent with previous reports.

**Supplementary information:**

The online version contains supplementary material available at 10.1007/s11657-026-01703-y.

## Introduction

Arterial hypertension and osteoporosis represent two of the most prevalent chronic disorders worldwide, affecting a significant portion of the global population [1, 2] and leading to a significant burden in terms of morbidity, mortality, and health-related costs [3–5].

These conditions are interrelated, with several studies demonstrating a higher risk of osteoporotic fractures in patients with hypertension than in those without [6–10]. Several hypotheses have been proposed to explain this increase in fracture risk in hypertensive patients, including alterations in calcium metabolism [11–14], enhanced sympathetic nervous system activity [15–18], dysregulation of the renin–angiotensin–aldosterone system (RAAS) [19–21], and microvascular disease [22–25].

While the evidence regarding increased fracture risk is relatively concordant, the effect of hypertension on bone mineral density (BMD) is less clear, with different studies reporting either a lower [11, 26], unaltered [27, 28], or even higher [29–31] BMD in hypertensive patients compared to normotensive controls. Moreover, the risk of fracture seems to be higher in hypertensive patients also after adjustment for bone density [10], thus suggesting that subtler changes in bone microarchitecture may be involved, not captured by BMD.

In recent years, various noninvasive methods for assessing bone microarchitectural quality have been developed and proposed; among them, the trabecular bone score (TBS) has emerged as one of the simplest, most accessible, and most informative [32, 33]. TBS is a texture-based metric that measures the rate of gray-level changes in lumbar spine dual-energy X-ray absorptiometry (DXA) images, providing an indirect estimation of bone microarchitectural health [32, 33]. Numerous studies have shown that TBS is able to predict the risk of incident fragility fractures independently from clinical risk factors and BMD [34, 35]; of note, the evaluation of TBS is of particular relevance in various settings of secondary osteoporosis, in which the risk of fractures is often less associated with BMD, but rather related to changes in bone microstructure and quality [36–40].

To date, data about the possible effects of hypertension on bone microarchitectural quality are scarce. Moreover, the in vivo effects of anti-hypertensive medications on bone quality are poorly explored. The primary aim of this study was thus to evaluate whether bone microarchitecture, non-invasively assessed by TBS, is altered in hypertensive patients. As a secondary aim, the association between the different classes of anti-hypertensive medications and TBS was also assessed.

## Methods

### Survey design and data collection

This study analyzes data from the 2005–2008 cycles of the National Health and Nutrition Examination Survey (NHANES), a cross-sectional survey program conducted in the U.S. by the National Center for Health Statistics (NCHS). The NHANES program is designed to include representative samples of the general, noninstitutionalized U.S. population of all age groups; to accomplish this objective, it employs a stratified, multistage, clustered probability sampling design, with oversampling of minorities such as non-Hispanic Black, Hispanic, and Asian persons, as well as people with low income and older adults. The survey involves a structured interview conducted in the homes of the participants, followed by a standardized health evaluation performed at a mobile examination center, which includes various laboratory tests and other examinations. A comprehensive description of the data collection methodology is reported on the NHANES website [41]. The original survey received approval from the Centers for Disease Control and Prevention Research Ethics Review Board. Written informed consent was obtained from all participants.

### Blood pressure measurements and definition of hypertension

Blood pressure measurements were conducted using calibrated mercury sphygmomanometers by trained health professionals, certified through a specific training program [41]. Participants were asked to sit quietly for at least 5 min before measurements were taken. Three consecutive blood pressure readings were recorded, with a fourth attempt being made if any of the previous readings were interrupted or incomplete. The average of the available readings was used as the representative value for systolic blood pressure and diastolic blood pressure. Hypertension was defined as systolic blood pressure ≥ 130 mmHg or diastolic blood pressure ≥ 80 mmHg, or currently taking blood pressure medications [42].

### Other clinical data and laboratory tests

Body measurements, including weight (kg), height (cm), and body mass index (BMI, kg/m^2^), were obtained during the mobile examination center visit. Information regarding menopausal status (in women), annual household income, current cigarette smoking, history of liver disease, recent hospitalization (within 1 year), and history of chronic glucocorticoid treatment for ≥ 3 months was based on self-report. Habitual levels of physical activity were assessed on a weekly/monthly basis using specific questionnaires; moderate activities were defined as those causing light sweating and/or a slight-to-moderate increase in breathing or heart rate for at least 10 min; vigorous activities were defined as those causing heavy sweating and/or a large increase in breathing or heart rate for at least 10 min. Dietary calcium intake was estimated based on a structured dietary interview. Ongoing pharmacological therapies were reported by each subject during the household interview, with direct verification of the medication containers by the interviewer whenever possible; data on specific dosage or previously discontinued prescriptions were not available. Laboratory methods for the measurement of all performed blood and urine tests are reported in detail on the NHANES website [41].

BMI categories were defined as follows: normal weight if BMI ≥ 18.5 kg/m^2^ and < 25 kg/m^2^; overweight if BMI ≥ 25 kg/m^2^ and < 30 kg/m^2^; obesity if BMI ≥ 30 kg/m^2^; underweight if BMI < 18.5 kg/m^2^. Diabetes mellitus was defined if any of the following conditions were met: (i) a fasting plasma glucose ≥ 126 mg/dL; (ii) a glycated hemoglobin (HbA1c) level ≥ 6.5% (48 mmol/mol); (iii) a self-reported diagnosis of diabetes; (iv) a self-reported use of antidiabetic drugs; subjects not meeting any of these criteria were considered non-diabetic, unless data were missing in all four. Estimated glomerular filtration rate (eGFR) was computed according to the Chronic Kidney Disease Epidemiology Collaboration (CKD-EPI) equation [43], and patients were stratified into three categories: eGFR ≥ 60 mL/min/1.73 m^2^, eGFR ≥ 30 to < 60 mL/min/1.73 m^2^, and eGFR < 30 mL/min/1.73 m^2^.

### BMD and TBS analysis

BMD was measured at the lumbar and femoral sites by DXA. All DXA examinations were performed by trained and certified radiology technologists. Scans were acquired on Hologic QDR-4500A fan-beam densitometers (Hologic, Inc., Bedford, Massachusetts) using software version Apex 3.0. Quality control phantoms were scanned daily to ensure accurate calibration of the densitometer. Further details of the DXA examination are documented on the NHANES website [41].

For each subject and at each skeletal site, we calculated *T*-scores as (BMD_subject_ − *µ*_reference_)/*σ*_reference_, where *µ*_reference_ and *σ*_reference_ are the mean and standard deviation of BMD in the reference group. As recommended by the World Health Organization (WHO) [44], the reference group for the calculation of *T*-scores at the total hip and femoral neck consisted of non-Hispanic white females 20 to 29 years of age from the NHANES III report [45]. For the calculation of *T*-score at the lumbar spine, the reference curves of the DXA manufacturer for 30-year-old white females were considered, taking into account the specific subset of vertebrae for which a valid BMD measurement was available.

TBS was extracted in adults aged 20 years or older from lumbar spine DXA images using a dedicated software (Med-Imap SA TBS Calculator version 2.1.0.2). In the calculation of TBS, BMI was used as an indirect measure of body thickness to improve the accuracy and comparability of TBS measurements across participants. Further details on TBS calculation are available on the NHANES website [41].

### Sample selection

**Fig. 1:**
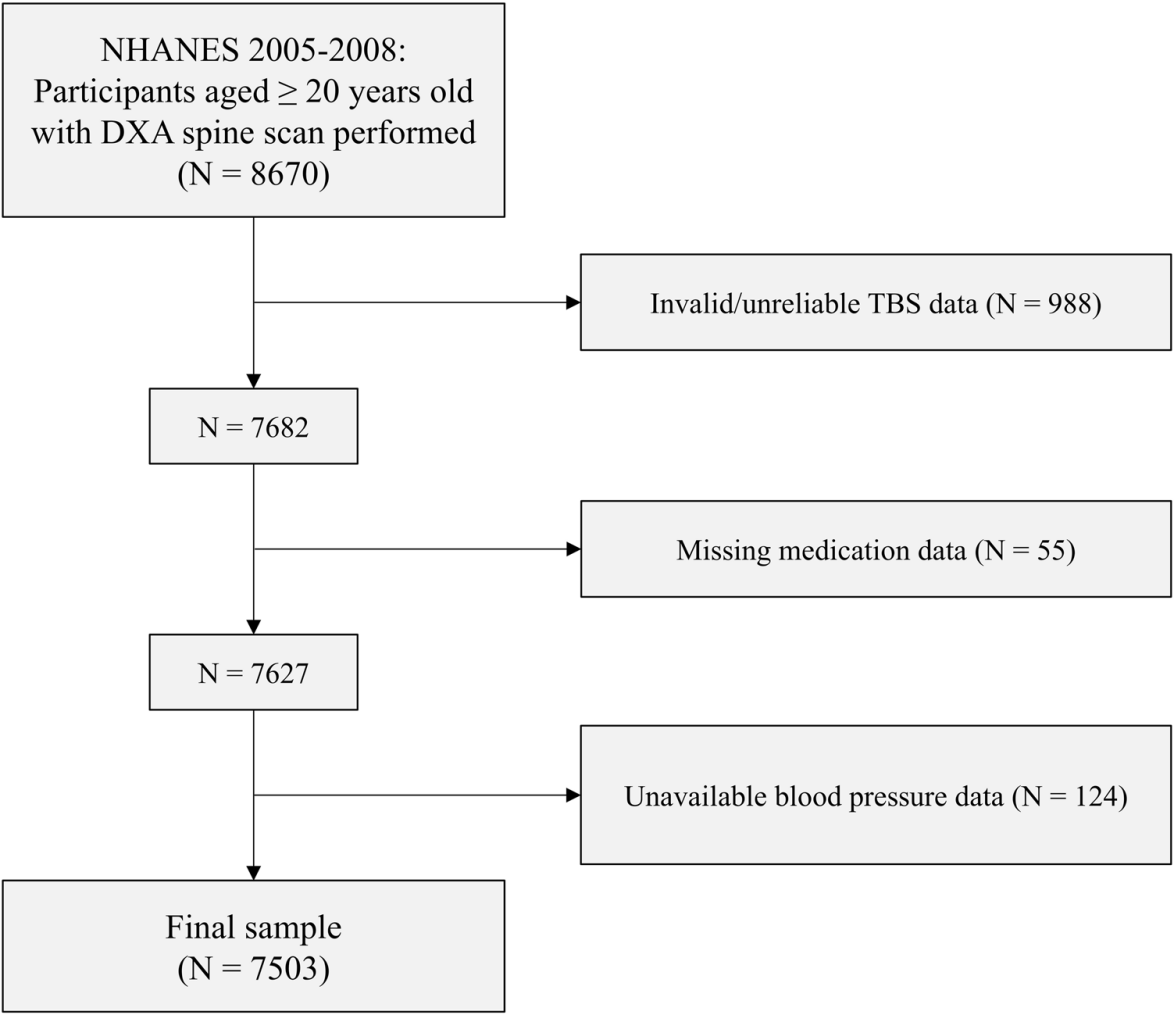
Flowchart of participant inclusion. DXA, dual-energy X-ray absorptiometry; NHANES, National Health and Nutrition Examination Survey; TBS, trabecular bone score

A total of 8670 subjects aged 20 years or older participated in the 2005–2008 NHANES survey cycles and had DXA spine scans performed. Among these, 988 subjects were excluded because of no valid/reliable TBS data (237 because of insufficient number of valid vertebrae for TBS estimation, 45 from missing BMI data, and 706 from BMI outside the range of 15 to 37 kg/m^2^, which represents the range within which TBS estimation is considered valid [33]). Of the remaining 7682 subjects, 55 were excluded because of incomplete medication data. Finally, 124 subjects were excluded because of unavailable blood pressure data, resulting in a final sample of 7503 subjects. The full process of sample selection is summarized graphically in Fig. 1.

### Statistical analysis

All analyses were conducted accounting for the complex survey design of NHANES, using appropriate weighting as suggested by the NCHS. Data were summarized as weighted means and standard deviation (SD) for continuous variables, and as weighted proportions for categorical data.

Multivariable linear regression analyses were performed to evaluate the association of hypertension and anti-hypertensive medications with TBS and BMD. Generalized additive models with spline smoothing were used to evaluate systolic and diastolic blood pressure as continuous predictors. All analyses were adjusted for relevant confounders, including age, sex, menopausal status, race/ethnicity, income, habitual physical activity, smoking status, BMI category, diabetes mellitus, eGFR category, history of liver disease, recent hospitalization (≤ 1 year), dietary calcium intake, 25OH-VitD levels, PPI treatment, hormone-blocking treatment, history of chronic GC treatment, and anti-hypertensive medications (ACE-inhibitors, ARBs, dihydropyridine CCBs, non-dihydropyridine CCBs, beta blockers, alpha blockers, loop diuretics, thiazide/thiazide-like diuretics, MRAs, other potassium-sparing diuretics, alpha-2 agonists, direct vasodilators). Sensitivity analyses were conducted excluding patients treated with anti-osteoporotic drugs.

Missing values were imputed by multiple imputations with chained equations, stratified by gender. To avoid bias, the imputation model included all covariates and outcomes used to fit the primary analysis models, as recommended [46, 47]. Fifty imputed datasets were created, and estimates were combined using Rubin’s rules [46]. In each analysis, subjects with missing outcomes were excluded.

A cut-off of 0.05 was adopted for the definition of statistical significance. Statistical analysis was performed using STATA 18 (StataCorp, College Station, Texas, USA) and R 4.4.0 (R Core Team, Vienna, Austria).

## Results

### Characteristics of the study population

Among the 7503 participants included in the analysis, 3586 subjects fulfilled the criteria for the diagnosis of arterial hypertension. Table 1 shows the clinical and biochemical characteristics of the study cohort, stratified by hypertension status. Among hypertensive patients, 1775 were treated with anti-hypertensive medications at the time of the examination. A descriptive summary of the anti-hypertensive medication classes prescribed in hypertensive patients is reported in Table 2.

**Table 1:** Descriptive characteristics of the study population stratified by the presence or absence of hypertension

Parameter	Subjects without hypertension (*N* = 3917)	Subjects with hypertension (*N* = 3586)	*p*-value
Age (years)	40.0 ± 11.0	55.1 ± 13.3	** < 0.001**
Sex (%)			** < 0.001**
Male	48.3	54.6	
Female	51.7	45.4	
Menopausal status, among females (%)^a^			** < 0.001**
Pre-menopausal	74.0	26.4	
Post-menopausal	26.0	73.6	
Race/ethnicity (%)			** < 0.001**
Non-Hispanic White	69.9	74.8	
Non-Hispanic Black	8.9	11.6	
Hispanic	14.7	9.0	
Other	6.5	4.6	
Annual household income (%)^b^			** < 0.001**
≥ 75,000 $	34.3	32.1	
$45,000–74,99	25.3	22.4	
$20,000–44,999	28.3	29.7	
< 20,000 $	12.1	15.8	
Habitual physical activity (%)			** < 0.001**
None	24.3	32.8	
Moderate	27.9	35.3	
Vigorous	47.8	31.9	
Smoking status (%) ^c^			** < 0.001**
Never smoker	52.6	48.6	
Former smoker	20.6	30.2	
Current smoker	26.8	21.2	
BMI category (%)			** < 0.001**
Normal weight	40.8	25.7	
Overweight	36.2	39.3	
Obesity	20.8	34.0	
Underweight	2.2	1.0	
Diabetes mellitus (%)	4.2	15.1	** < 0.001**
eGFR category (%) ^d^			** < 0.001**
≥ 60 mL/min/1.73 m^2^	97.7	87.5	
≥ 30 to < 60 mL/min/1.73 m^2^	2.2	11.5	
< 30 mL/min/1.73 m^2^	0.1	1.0	
History of liver disease (%)^e^	2.7	3.9	**0.033**
Recent hospitalization (≤ 1 year) (%)^f^	6.9	11.9	** < 0.001**
Dietary calcium intake (g/day)^g^	0.98 ± 0.48	0.92 ± 0.51	**0.001**
25OH-VitD (ng/mL)^h^	26.5 ± 7.3	26.0 ± 7.9	0.107
PPI treatment (%)	5.0	12.0	** < 0.001**
Hormone-blocking treatment (%)	0.4	0.4	0.741
History of chronic GC treatment (%)^i^	2.3	3.4	**0.016**

Significant *p*-values are highlighted in bold

*25OH-VitD* 25-hydroxyvitamin D, *BMI* body mass index, *eGFR* estimated glomerular filtration rate, *GC* glucocorticoid, *PPIs* proton pump inhibitors

^a^Missing data in 207 (5.8%) female subjects

^b^Missing data in 239 (3.2%) subjects

^c^Missing data in 5 (0.1%) subjects

^d^Missing data in 319 (4.3%) subjects

^e^Missing data in 13 (0.2%) subjects

^f^Missing data in 3 (< 0.1%) subjects

^g^Missing data in 152 (2.0%) subjects

^h^Missing data in 803 (10.7%) subjects

^i^Missing data in 59 (0.8%) subjects

**Table 2:** Summary of the anti-hypertensive medication classes prescribed in hypertensive patients (*N* = 3586)

Anti-hypertensive medication class	Hypertensive patients treated	
	Absolute number, *N*	Weighted percentage, %^a^
ACE-inhibitors	784	18.8
ARBs	430	10.8
Dihydropyridine CCBs	453	10.2
Non-dihydropyridine CCBs	139	3.4
Beta blockers	743	19.0
Alpha blockers	89	1.7
Loop diuretics	165	3.4
Thiazide(-like) diuretics	672	16.7
MRAs	37	0.9
Other potassium-sparing diuretics	138	3.4
Alpha-2 agonists	36	0.7
Direct vasodilators	17	0.3

*ACE* angiotensin-converting enzyme, *ARBs* angiotensin II receptor blockers, *CCBs* calcium channel blockers, *MRAs* mineralocorticoid receptor antagonists, *NCHS* National Center for Health Statistics

^a^Weighted percentages are computed in the sub-cohort of hypertensive patients (*N* = 3586), in accordance with the survey design, using appropriate weighting as suggested by the NCHS

### Association between hypertension and bone outcomes

**Fig. 2:**
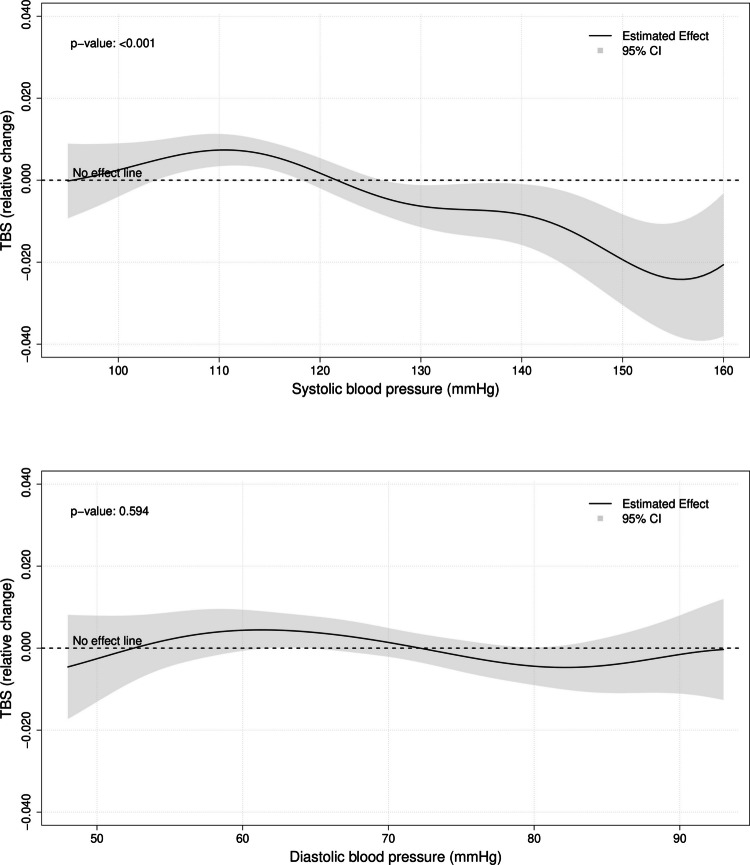
Generalized additive models with spline smoothing evaluating the association of systolic (upper panel) and diastolic (lower panel) blood pressure with TBS. To avoid the possible interference of anti-hypertensive medications, the analysis was limited to untreated hypertensive and normotensive subjects, as in the primary model (Table 3, Model 1A). All analyses were adjusted for the same set of covariates as in the primary model. The p-values summarize the statistical significance of the contribution of systolic and diastolic blood pressure to the outcome, relative to the null hypothesis of no meaningful relation. BMD, bone mineral density; CI, confidence interval; TBS, trabecular bone score

The association between hypertension and bone outcomes was first evaluated excluding from the analysis treated hypertensive patients, to avoid the possible interference of anti-hypertensive medications. Hypertension was independently associated with degraded bone microarchitecture, as demonstrated by lower TBS values (*β* = −0.010; 95%CI, [−0.016, −0.003]; *p* = 0.008) (Table 3, Model 1A); on the contrary, no association was observed between hypertension and BMD *T*-scores at lumbar spine (*β* =  + 0.04; 95%CI, [− 0.05, + 0.13]; *p* = 0.362), total hip (*β* = −0.02; 95%CI, [− 0.10, + 0.05]; *p* = 0.481), or femoral neck (*β* = −0.03; 95%CI, [−0.10, + 0.03]; *p* = 0.298) (Table 3, Model 1A). The association between hypertension and TBS remained statistically significant even after further adjusting the multivariable regression analysis for BMD at lumbar spine and femoral neck (*β* = −0.011; 95%CI, [−0.017, −0.005]; *p* = 0.001) (Table 3, Model 1B). No significant effect modification was observed according to sex (*p*-value for interaction > 0.05 in all analyses). When evaluating blood pressure as a quantitative measure, a significant inverse relationship was observed between systolic blood pressure and TBS values (*p* < 0.001); conversely, no correlation was found for diastolic blood pressure (*p* = 0.594) (Fig. 2).

**Table 3:** Multivariable linear regression models evaluating the effect of hypertension on TBS and on BMD *T*-scores at lumbar spine, total hip and femoral neck

Model	Lumbar spine TBS			Lumbar spine *T*-score			Total hip *T*-score^a^			Femoral neck *T*-score^a^		
	*β*-coeff	95%CI	*p*-value	*β*-coeff	95%CI	*p*-value	*β*-coeff	95%CI	*p*-value	*β*-coeff	95%CI	*p*-value
Untreated hypertensive subjects compared to normotensive subjects (*N* = 5728)												
Model 1A^b^	−0.010	(− 0.016, −0.003)	**0.008**	+ 0.04	(−0.05, + 0.13)	0.362	−0.02	(−0.10, + 0.05)	0.481	−0.03	(−0.10, + 0.03)	0.298
Model 1B^c^	−0.011	(−0.017, −0.005)	**0.001**	-	-	-	-	-	-	-	-	-
All hypertensive subjects compared to normotensive subjects (*N* = 7503)												
Model 2A^d^	−0.010	(−0.017, −0.004)	**0.002**	+ 0.05	(−0.03, + 0.14)	0.182	−0.02	(−0.08, + 0.04)	0.462	−0.03	(−0.08, + 0.03)	0.353
Model 2B^e^	−0.012	(−0.018, −0.006)	** < 0.001**	-	-	-	-	-	-	-	-	-

Significant *p*-values are highlighted in bold

*25OH-VitD* 25-hydroxyvitamin D, *ACE* angiotensin-converting enzyme, *ARBs* angiotensin II receptor blockers, *BMI* body mass index, *CCBs* calcium channel blockers, *CI* confidence interval, *eGFR* estimated glomerular filtration rate, *GC* glucocorticoid, *MRAs* mineralocorticoid receptor antagonists, *PPIs* proton pump inhibitors, *TBS* trabecular bone score, *β-coeff β*-coefficient

^a^Missing outcome data in 174 (3.0%) subjects for Models 1A-1B, and in 303 (4.0%) subjects for Models 2A-2B

^b^Adjusted for age, sex, menopausal status, race/ethnicity, income, habitual physical activity, smoking status, BMI category, diabetes mellitus, eGFR category, history of liver disease, recent hospitalization (≤ 1 year), dietary calcium intake, 25OH-VitD levels, PPI treatment, hormone-blocking treatment, and history of chronic GC treatment

^c^Adjusted for the same covariates as Model 1A, plus lumbar spine *T*-score and femoral neck *T*-score

^d^Adjusted for the same covariates as Model 1A, plus current use of ACE-inhibitors, ARBs, dihydropyridine CCBs, non-dihydropyridine CCBs, beta blockers, alpha blockers, loop diuretics, thiazide(-like) diuretics, MRAs, other potassium-sparing diuretics, alpha-2 agonists and direct vasodilators

^e^Adjusted for the same covariates as Model 2A, plus lumbar spine *T*-score and femoral neck *T*-score

Sensitivity analyses were conducted, with no relevant changes in the results. A degradation of bone quality, without significant variation in bone mass, was confirmed when extending the analysis to all hypertensive patients (both treated and untreated), with further adjustment for the anti-hypertensive medications used (Table 3, Models 2A and 2B). The same results were observed when excluding from the analyses all patients actively treated with anti-osteoporotic drugs (Supplementary Table 1).

As ancillary findings, the role of other known parameters as predictors of bone outcomes was confirmed. The full results regarding the relationships between the covariates used in the regression models and bone outcomes are reported in Supplementary Table 2.

### Association between anti-hypertensive medications and bone outcomes

The association between anti-hypertensive medications and bone outcomes was evaluated among hypertensive patients (*N* = 3586). None of the anti-hypertensive medication classes showed a significant association with TBS (Table 4). Increased BMD was observed in patients treated with angiotensin II receptor blockers (ARBs) at all skeletal sites, and in patients treated with thiazides at the lumbar spine (Table 4). Decreased BMD was observed in patients treated with dihydropyridine calcium channel blockers (CCBs) at the total hip and femoral neck, and in patients treated with loop diuretics at the total hip (Table 4). The same results were confirmed when excluding from the analyses all patients actively treated with anti-osteoporotic drugs (Supplementary Table 3). Of note, no association was found between the number of antihypertensive medications and either BMD or TBS (*p* > 0.05 for all bone outcomes).

**Table 4:** Multivariable linear regression models evaluating the effect of anti-hypertensive medications on TBS and on BMD *T*-scores at lumbar spine, total hip and femoral neck, among hypertensive patients (*N* = 3586)

Parameter	Lumbar spine TBS			Lumbar spine *T*-score			Total hip *T*-score^a^			Femoral neck *T*-score^a^		
	*β*-coeff	95%CI	*p*-value	*β*-coeff	95%CI	*p*-value	*β*-coeff	95%CI	*p*-value	*β*-coeff	95%CI	*p*-value
ACE-inhibitors	+ 0.000	(−0.011, + 0.012)	0.939	+ 0.11	(−0.06, + 0.28)	0.203	+ 0.12	(−0.02, + 0.27)	0.089	+ 0.10	(−0.04, + 0.24)	0.157
ARBs	+ 0.005	(−0.009, + 0.018)	0.483	+ 0.30	(+ 0.11, + 0.48)	**0.003**	+ 0.24	(+ 0.05, + 0.43)	**0.014**	+ 0.20	(+ 0.05, + 0.36)	**0.012**
Dihydropyridine CCBs	−0.011	(−0.025, + 0.003)	0.112	−0.07	(−0.23, + 0.09)	0.379	−0.13	(−0.25, −0.00)	**0.044**	−0.16	(−0.24, −0.07)	**0.001**
Non-dihydropyridine CCBs	−0.023	(−0.048, + 0.001)	0.057	−0.10	(−0.32, + 0.12)	0.350	−0.14	(−0.38, + 0.10)	0.233	−0.02	(−0.19, + 0.14)	0.752
Beta blockers	+ 0.002	(−0.010, + 0.014)	0.760	+ 0.07	(−0.08, + 0.22)	0.327	+ 0.05	(−0.08, + 0.17)	0.447	+ 0.02	(−0.08, + 0.11)	0.739
Alpha blockers	+ 0.017	(−0.008, + 0.042)	0.176	+ 0.18	(−0.21, + 0.56)	0.362	−0.07	(−0.39, + 0.25)	0.675	−0.12	(−0.40, + 0.17)	0.410
Loop diuretics	−0.028	(−0.073, + 0.016)	0.195	−0.00	(−0.46, + 0.46)	0.997	−0.34	(−0.63, −0.06)	**0.020**	−0.17	(−0.41, + 0.07)	0.164
Thiazide(-like) diuretics	+ 0.011	(−0.003, + 0.025)	0.129	+ 0.27	(+ 0.11, + 0.42)	**0.001**	+ 0.03	(−0.11, + 0.17)	0.647	+ 0.03	(−0.08, + 0.14)	0.543
MRAs	+ 0.018	(−0.029, + 0.066)	0.435	+ 0.26	(−0.31, + 0.84)	0.358	−0.00	(−0.59, + 0.58)	0.987	−0.03	(−0.49, + 0.43)	0.897
Other potassium-sparing diuretics	−0.006	(−0.038, + 0.026)	0.713	−0.05	(−0.37, + 0.26)	0.731	+ 0.11	(−0.09, + 0.32)	0.278	+ 0.07	(−0.11, + 0.25)	0.460
Alpha-2 agonists	−0.041	(−0.130, + 0.048)	0.355	−0.09	(−0.94, + 0.76)	0.829	+ 0.02	(−0.63, + 0.68)	0.943	−0.15	(−0.69, + 0.40)	0.585
Direct vasodilators	+ 0.033	(−0.045, + 0.111)	0.394	+ 0.05	(−0.72, + 0.82)	0.893	+ 0.08	(−0.62, + 0.78)	0.813	+ 0.29	(−0.48, + 1.07)	0.448

Medications are modeled as binary variables. All analyses are adjusted for age, sex, menopausal status, race/ethnicity, income, habitual physical activity, smoking status, BMI category, diabetes mellitus, eGFR category, history of liver disease, recent hospitalization (≤ 1 year), dietary calcium intake, 25OH-VitD levels, PPI treatment, hormone-blocking treatment, and history of chronic GC treatment

Significant *p*-values are highlighted in bold

*ACE*, angiotensin-converting enzyme; *ARBs*, angiotensin II receptor blockers; *CCBs*, calcium channel blockers; *CI*, confidence interval; *MRAs*, mineralocorticoid receptor antagonists; *PPIs*, proton-pump inhibitors; *β-coeff*, β-coefficient

^a^Missing outcome data in 211 (5.9%) subjects

## Discussion

In the present study, we evaluated the association between arterial hypertension and TBS in a large community-based sample extracted from the general U.S. population. Our findings revealed a significant impairment of bone quality among hypertensive patients compared to their normotensive counterparts. Conversely, no association was found between hypertension and BMD at any site. Notably, the association between hypertension and lower TBS values persisted even after adjustment for bone density, further supporting the hypothesis that hypertensive patients may have a distinct impairment in bone microarchitectural quality that is independent of BMD. The association between hypertension and degraded bone quality was consistent across a range of sensitivity analyses and was observed irrespective of the inclusion/exclusion of patients treated with antihypertensive medications; this is noteworthy because it supports a direct link between blood pressure elevation and bone microarchitectural degradation, independent of the effect of anti-hypertensive therapies. Moreover, in the sub-cohort of untreated individuals, systolic blood pressure demonstrated an approximately linear inverse relationship with TBS values, suggesting the presence of a *dose–response* relationship between the exposure and the outcome.

Over the last three decades, several studies have shown that hypertension is an independent risk factor for osteoporotic fractures [6–10]; although the precise pathophysiological mechanisms still need to be fully elucidated, several potential pathways have been suggested [48–50]. For example, hypertension is associated with an increased urinary calcium excretion, which promotes an increase in PTH secretion and bone turnover [11–14]. Moreover, both osteoblasts and osteoclasts express adrenergic receptors, suggesting that the hyperactivation of the sympathetic nervous system may also play a role [15–18]; experimental studies have reported that adrenergic stimulation can increase osteoclast and inhibit osteoblast function, with a negative impact on bone turnover [15–18]. The dysregulation of the RAAS may also exert detrimental effects on bone health; both osteoclasts and osteoblasts express angiotensin and mineralocorticoid receptors, and RAAS activation overall favors bone resorption over bone formation [19–21]. Finally, there is evidence suggesting a direct association between arterial stiffness, microvascular disease, and degraded bone quality and strength, possibly mediated by a decreased blood flow to the bone, which might impair its remodeling processes and microarchitecture [22–25].

Overall, these mechanisms demonstrate a relevant pathophysiological connection between arterial hypertension and bone metabolism, which could account for the higher fracture incidence observed in hypertensive patients. However, although the evidence regarding increased fracture risk is relatively concordant [6–10], the relationship between hypertension and BMD remains less clear, with different studies reporting either a lower [11, 26], unaltered [27, 28], or even higher [29–31] BMD in hypertensive patients compared to normotensive controls. Moreover, as noted earlier, there is evidence indicating that the risk of fracture is higher in hypertensive patients even after adjustment for bone density [10], thus suggesting that other factors are probably also at play in determining the increased bone fragility observed in hypertensive patients.

The field of osteoporosis has evolved significantly in recent years, with increasing emphasis on indices of bone quality as a complementary measure in the assessment of fracture risk [32, 33]. In this context, TBS has emerged as one of the simplest, most accessible, and most informative [32, 33], demonstrating an independent diagnostic and prognostic value across several settings of primary and secondary osteoporosis [34–40].

The association between hypertension and bone microarchitectural quality, however, remains poorly explored, and published data on this topic are limited. An inverse association between hypertension and TBS was first reported by Shih et al. [51]. More recently, Lee et al. observed similar results in postmenopausal women, in the specific setting of low-renin hypertension [52]. However, although these studies provided interesting insights about the possible impact of hypertension on bone microarchitecture, they had some limitations. In particular, in both cases, the adjustment of the analyses was limited to a small number of potential confounders; none of the two studies accounted for the possible specific role of the different classes of anti-hypertensive medications, although many of these medications have relevant and distinct effects on bone metabolism; none of the two studies reported data about untreated hypertensive patients. In our study, many of these issues were addressed. The analyses were adjusted for a wider range of confounding factors, including all classes of anti-hypertensive medications. A specific evaluation limited to untreated hypertensive patients was also performed.

In addition, in our study, we also evaluated the association between each class of anti-hypertensive medications and TBS. No significant relationship was found. Conversely, various associations between anti-hypertensive medications and BMD were present, aligning with previous reports.

In literature, thiazide and loop diuretics are the two anti-hypertensive medication classes showing the most consistent association with bone outcomes. Thiazide diuretics have been widely associated with a positive effect on BMD [53–56] and a decrease in fracture risk [57–61], through both a reduction of urinary calcium excretion and direct effects on bone [48, 62, 63]. To date, only one study has assessed their association with TBS in an independent cohort [53], reporting non-significant results, as in our analysis. On the opposite, loop diuretics have been widely associated with a negative effect on BMD [64, 65] and an increase in fracture risk [60, 66, 67], through both an increase of increasing urinary calcium loss and—for fractures—of the risk of falls [48, 62, 63]. To date, only one study has assessed their association with TBS, reporting lower TBS values in past, but not current, loop diuretic users [64].

Among drugs acting on RAAS, the most consistent evidence is available for ARBs, for which a beneficial effect on bone outcomes has been demonstrated in various independent cohorts [68–71], probably through a counteraction of the harmful effects of RAAS on bone [48, 62, 63]. Conversely, either a beneficial, neutral, or even detrimental effect has been reported for ACE-inhibitors (ACEi) [56, 68–74], while evidence for mineralocorticoid receptor antagonists (MRAs) is scarce [75, 76]. To date, only two studies have assessed the relationship between the use of ARBs/ACEi and TBS [77, 78]: no association was observed with ARB use, while a negative association was reported for ACEi in women; nevertheless, the strength of this latter conclusion was limited by the low sample size (less than 50 women treated with ACEi). To our knowledge, no study has ever assessed the association between MRAs and TBS.

With regard to the effects on bone of other anti-hypertensive medication classes, literature is inconsistent. Either a beneficial, neutral, or detrimental effect in terms of BMD and/or fracture risk has been reported for CCBs [60, 79–81] and beta blockers [82–86]. To our knowledge, no study has ever assessed their association with TBS. Alpha blockers have been linked to higher fracture risk, possibly due to an increased risk of hypotension and falls [87, 88]; however, whether they have a direct impact on bone metabolism in vivo remains unclear.

The main strength of this study is that it is conducted on a large, unselected, community-based sample extracted from the general U.S. population; its stratified, multistage, clustered probability design with sample weighting minimizes the effects of selection bias and survey non-response, enhancing the generalizability of the results. In addition, the large sample size and the availability of extensive clinical and biochemical data allowed the analysis to be adjusted for a wide range of possible confounders. Moreover, the presence of a relevant proportion of untreated hypertensive patients allowed us to perform a specific analysis in this subgroup, which demonstrated a significant association between blood pressure elevation and bone microarchitectural degradation in the absence of the interfering effect of anti-hypertensive therapies.

Our study also has limitations. First, its cross-sectional design does not allow the demonstration of cause-effect relationships and temporal trends; this is particularly relevant when evaluating the association with anti-hypertensive medications; nevertheless, despite this limitation, the observed relationships between anti-hypertensive medications and BMD were consistent with the existing literature, thus suggesting that, if any drug class had a major effect on TBS, this would probably have been detected. Second, residual confounding cannot be excluded; NHANES did not provide sufficiently detailed or consistently available information on several potentially relevant variables, including hypertension duration, fall risk, some bone-active comorbidities, lifestyle factors, and vitamin D or calcium supplementation; although some of these factors were partly addressed in the models, incomplete adjustment may still have influenced our findings. Third, data on medication dosages or on previously discontinued prescriptions were not available; therefore, the possible presence of a dose–response relationship between anti-hypertensive medications and bone outcomes could not be investigated, nor could the effect of previous use. Fourth, fracture outcomes were not analyzed; although NHANES includes self-reported prior fractures, these data are recall-based, do not capture unrecognized fractures, and yielded too few osteoporotic fracture events for robust analysis; therefore, our findings should be interpreted as showing an association between hypertension and impaired bone microarchitecture, rather than direct evidence of increased fracture risk. Finally, the available data lacked sufficient detail to distinguish essential from secondary hypertension; some conditions causing secondary hypertension (e.g., Cushing’s syndrome, primary aldosteronism, or pheochromocytoma) may independently contribute to bone damage, regardless of blood pressure values; however, at a population level, they account for a minority of hypertension cases, and their impact on the overall study results—if any—is thus likely to be minimal.

In conclusion, our study shows an independent association between arterial hypertension and impaired bone quality, non-invasively assessed by TBS. Notably, we reported this association also in untreated hypertensive patients, therefore excluding the confounding effect of anti-hypertensive medications. On the other hand, no significant association was found between hypertension and BMD, suggesting that, in hypertensive patients, bone microarchitecture might be more strongly impaired than bone mass. With regard to anti-hypertensive medications, some classes demonstrated a significant association with BMD, consistent with previous reports; on the contrary, none exhibited a significant association with TBS values. Overall, these results provide further insight into the relationship between hypertension and bone health; the specific degradation of bone microarchitecture, independent of bone density, may help explain the increased skeletal fragility that characterizes hypertensive patients, which cannot be justified solely by BMD. Considering the global prevalence of hypertension and osteoporosis, refining the knowledge of the pathophysiological mechanisms linking these two conditions could have significant implications for risk stratification, management strategies and therapeutic interventions at a population level.

## Supplementary Information

Below is the link to the electronic supplementary material.

ESM 1 (PDF 322 KB)

## Data Availability

All data analyzed during this study are publicly available at https://wwwn.cdc.gov/nchs/nhanes/Default.aspx.
