# Supplementary material for: Bone microarchitectural degradation in hypertensive patients: a population-based study
Source: Arch Osteoporos. 2026 Apr 27;21(1):73. doi: 10.1007/s11657-026-01703-y (PMC13121177; doi:10.1007/s11657-026-01703-y)
Supplement: Supplementary file 1 — (PDF 322 KB) [file 11657_2026_1703_MOESM1_ESM.pdf]

SUPPLEMENTARY MATERIAL

Bone microarchitectural degradation in hypertensive patients: a population-based study

Fabio Bioletto[1], Martina Bollati[1], Marco Barale[2], Chiara Lopez[1], Alessia Pusterla[1], Emanuela Arvat[2], Ezio Ghigo[1], Mauro Maccario[1], Massimo Procopio[1], Mirko Parasiliti-Caprino[1]

[1] Division of Endocrinology, Diabetes and Metabolism; Department of Medical Sciences; University of Turin; Turin, Italy.

[2] Division of Oncological Endocrinology; Department of Medical Sciences; University of Turin; Turin, Italy.

Supplementary Table 1. Multivariable linear regression models evaluating the effect of hypertension on TBS and on BMD T-scores at lumbar spine, total hip and femoral neck, restricted to subjects not currently treated with anti-osteoporotic drugs. The excluded subjects were 115 in Models 1A-1B (95 on bisphosphonates, 19 on raloxifene, 1 on teriparatide, none on other treatments), and 224 in Models 2A-2B (186 on bisphosphonates, 37 on raloxifene, 1 on teriparatide, none on other treatments). Significant p-values are highlighted in bold. Abbreviations: 25OH-VitD, 25-hydroxyvitamin D; ACE, angiotensin-converting enzyme; ARBs, angiotensin II receptor blockers; BMI, body mass index; CCBs, calcium channel blockers; CI, confidence interval; eGFR, estimated glomerular filtration rate; GC, glucocorticoid; MRAs, mineralocorticoid receptor antagonists; PPIs, proton-pump inhibitors; TBS, trabecular bone score; β-coeff, β-coefficient.

**Model**	**Lumbar spine TBS**			**Lumbar spine T-score**			**Total hip T-score[a]**			**Femoral neck T-score[a]**		
	**β-coeff**	**95%CI**	**p-value**	**β-coeff**	**95%CI**	**p-value**	**β-coeff**	**95%CI**	**p-value**	**β-coeff**	**95%CI**	**p-value**
**Untreated hypertensive subjects compared to normotensive subjects (N = 5613)**												
Model 1A[b]	-0.010	(-0.017, -0.003)	**0.009**	+0.04	(-0.05, +0.13)	0.374	-0.03	(-0.10, +0.05)	0.457	-0.04	(-0.11, +0.03)	0.296
Model 1B[c]	-0.010	(-0.018, -0.005)	**0.001**	-	-	-	-	-	-	-	-	-
**All hypertensive subjects compared to normotensive subjects (N = 7279)**												
Model 2A[d]	-0.011	(-0.017, -0.004)	**0.002**	+0.05	(-0.04, +0.14)	0.233	-0.03	(-0.09, +0.03)	0.330	-0.03	(-0.09, +0.02)	0.231
Model 2B[e]	-0.012	(-0.019, -0.006)	**<0.001**	-	-	-	-	-	-	-	-	-

[a] Missing outcome data in 167 (3.0%) subjects for Models 1A-1B, and in 285 (3.9%) subjects for Models 2A-2B

[b] Adjusted for age, sex, menopausal status, race/ethnicity, income, habitual physical activity, smoking status, BMI category, diabetes mellitus, eGFR category, history of liver disease, recent hospitalization (≤ 1 year), dietary calcium intake, 25OH-VitD levels, PPI treatment, hormone-blocking treatment, and history of chronic GC treatment

[c] Adjusted for the same covariates as Model 1A, plus lumbar spine T-score and femoral neck T-score

[d] Adjusted for the same covariates as Model 1A, plus current use of ACE-inhibitors, ARBs, dihydropyridine CCBs, non-dihydropyridine CCBs, beta blockers, alpha blockers, loop diuretics, thiazide(-like) diuretics, MRAs, other potassium-sparing diuretics, alpha-2 agonists and direct vasodilators

[e] Adjusted for the same covariates as Model 2A, plus lumbar spine T-score and femoral neck T-score

Supplementary Table 2. Complete results of the primary analysis evaluating the effect of hypertension on bone outcomes among untreated hypertensive and normotensive subjects (Table 3, Model 1A). Significant p-values are highlighted in bold. Abbreviations: 25OH-VitD, 25-hydroxyvitamin D; BMI, body mass index; CI, confidence interval; eGFR, estimated glomerular filtration rate; GC, glucocorticoid; ref, reference; PPIs, proton pump inhibitors; TBS, trabecular bone score; β-coeff, β-coefficient.

**Parameter**	**Lumbar spine TBS**			**Lumbar spine T-score**			**Total hip T-score**			**Femoral neck T-score**		
	**β-coeff**	**95%CI**	**p-value**	**β-coeff**	**95%CI**	**p-value**	**β-coeff**	**95%CI**	**p-value**	**β-coeff**	**95%CI**	**p-value**
Hypertension	-0.010	(-0.016, -0.003)	**0.008**	+0.04	(-0.05, +0.13)	0.362	-0.02	(-0.10, +0.05)	0.481	-0.03	(-0.10, +0.03)	0.298
Age (per 10 years increase)	-0.030	(-0.032, -0.028)	**<0.001**	-0.05	(-0.07, -0.02)	**0.001**	-0.18	(-0.20, -0.16)	**<0.001**	-0.30	(-0.32, -0.28)	**<0.001**
Sex and menopausal status												
Male	0 (ref)			0 (ref)			0 (ref)			0 (ref)		
Female, pre-menopausal	+0.038	(+0.029, +0.047)	**<0.001**	+0.15	(+0.08, +0.23)	**<0.001**	-0.66	(-0.74, -0.59)	**<0.001**	-0.37	(-0.43, -0.30)	**<0.001**
Female, post-menopausal	-0.014	(-0.025, -0.004)	**0.010**	-0.84	(-0.98, -0.69)	**<0.001**	-1.06	(-1.17, -0.96)	**<0.001**	-0.62	(-0.72, -0.51)	**<0.001**
Race/ethnicity												
Non-Hispanic White	0 (ref)			0 (ref)			0 (ref)			0 (ref)		
Non-Hispanic Black	+0.005	(-0.004, +0.014)	0.241	+0.65	(+0.55, +0.75)	**<0.001**	+0.67	(+0.55, +0.78)	**<0.001**	+0.71	(+0.61, +0.81)	**<0.001**
Hispanic	-0.003	(-0.011, +0.004)	0.367	-0.22	(-0.35, -0.10)	**0.001**	+0.11	(+0.02, +0.21)	**0.023**	+0.14	(+0.05, +0.24)	**0.005**
Other	-0.009	(-0.026, +0.007)	0.259	-0.20	(-0.37, -0.02)	**0.028**	-0.00	(-0.14, +0.13)	0.962	-0.00	(-0.13, +0.12)	0.950
Annual household income												
≥ 75,000 $	0 (ref)			0 (ref)			0 (ref)			0 (ref)		
45,000-74,999 $	-0.004	(-0.013, +0.005)	0.398	+0.00	(-0.10, +0.10)	0.982	-0.07	(-0.14, +0.01)	0.079	-0.07	(-0.16, +0.01)	0.093
20,000-44,999 $	-0.012	(-0.020, -0.004)	**0.003**	-0.14	(-0.24, -0.03)	**0.014**	-0.11	(-0.21, -0.00)	**0.040**	-0.05	(-0.15, +0.05)	0.314
<20,000 $	-0.017	(-0.024, -0.009)	**<0.001**	-0.19	(-0.32, -0.06)	**0.004**	-0.15	(-0.26, -0.04)	**0.011**	-0.10	(-0.21, +0.01)	0.068
Habitual physical activity												
None	0 (ref)			0 (ref)			0 (ref)			0 (ref)		
Moderate	+0.006	(-0.002, +0.013)	0.123	+0.01	(-0.11, +0.12)	0.920	+0.03	(-0.07, +0.13)	0.569	-0.02	(-0.12, +0.07)	0.635
Vigorous	+0.012	(+0.005, +0.020)	**0.003**	+0.16	(+0.05, +0.26)	**0.005**	+0.20	(+0.12, +0.29)	**<0.001**	+0.16	(+0.07, +0.25)	**0.001**
Smoking status												
Never smoker	0 (ref)			0 (ref)			0 (ref)			0 (ref)		
Former smoker	-0.004	(-0.015, +0.006)	0.415	+0.03	(-0.07, +0.14)	0.539	-0.03	(-0.12, +0.07)	0.525	-0.03	(-0.13, +0.06)	0.479
Current smoker	-0.015	(-0.023, -0.007)	**0.001**	+0.00	(-0.08, +0.09)	0.926	-0.08	(-0.16, -0.01)	**0.033**	-0.02	(-0.10, +0.07)	0.708
BMI category												
Normal weight	0 (ref)			0 (ref)			0 (ref)			0 (ref)		
Overweight	-0.025	(-0.032, -0.018)	**<0.001**	+0.42	(+0.35, +0.49)	**<0.001**	+0.56	(+0.49, +0.63)	**<0.001**	+0.46	(+0.39, +0.52)	**<0.001**
Obesity	-0.104	(-0.112, -0.095)	**<0.001**	+0.70	(+0.59, +0.82)	**<0.001**	+1.02	(+0.92, +1.11)	**<0.001**	+0.88	(+0.80, +0.96)	**<0.001**
Underweight	-0.013	(-0.032, +0.005)	0.152	-0.51	(-0.80, -0.22)	**0.001**	-0.74	(-0.95, -0.53)	**<0.001**	-0.51	(-0.75, -0.28)	**<0.001**
Diabetes mellitus	-0.017	(-0.029, -0.004)	**0.010**	+0.03	(-0.11, +0.17)	0.678	-0.04	(-0.16, +0.09)	0.563	+0.00	(-0.13, +0.13)	0.968
eGFR category												
≥ 60 mL/min/1.73 m^2	0 (ref)			0 (ref)			0 (ref)			0 (ref)		
≥ 30 to <60 mL/min/1.73 m^2	+0.012	(-0.008, +0.033)	0.216	+0.03	(-0.22, +0.27)	0.831	-0.14	(-0.33, +0.05)	0.151	+0.00	(-0.18, +0.19)	0.960
<30 mL/min/1.73 m^2	-0.006	(-0.070, +0.058)	0.848	-0.18	(-0.99, +0.63)	0.643	-0.19	(-0.78, +0.41)	0.522	+0.04	(-0.55, +0.63)	0.887
History of liver disease	-0.012	(-0.027, +0.003)	0.106	-0.08	(-0.25, +0.08)	0.294	-0.02	(-0.12, +0.09)	0.723	-0.08	(-0.18, +0.03)	0.156
Recent hospitalization (≤ 1 year)	+0.005	(-0.007, +0.017)	0.395	+0.01	(-0.13, +0.14)	0.939	-0.03	(-0.13, +0.07)	0.490	-0.01	(-0.12, +0.10)	0.866
Dietary calcium intake (per 1 g/day increase)	+0.005	(+0.000, +0.009)	**0.038**	+0.04	(-0.02, +0.09)	0.211	+0.07	(+0.03, +0.11)	**0.001**	+0.07	(+0.03, +0.12)	**0.001**

25OH-VitD (per 10 ng/mL increase)	+0.008	(+0.004, +0.011)	**<0.001**	+0.05	(+0.00, +0.10)	**0.040**	+0.08	(+0.04, +0.12)	**0.001**	+0.07	(+0.03, +0.11)	**0.001**
PPI treatment	-0.022	(-0.036, -0.009)	**0.002**	-0.22	(-0.42, -0.02)	**0.030**	-0.11	(-0.24, +0.01)	0.081	-0.13	(-0.25, -0.02)	**0.026**
Hormone-blocking treatment	+0.004	(-0.029, +0.037)	0.806	+0.19	(-0.46, +0.83)	0.558	+0.16	(-0.37, +0.69)	0.546	+0.05	(-0.68, +0.78)	0.885
History of chronic GC treatment	-0.004	(-0.026, +0.018)	0.729	+0.08	(-0.12, +0.27)	0.431	-0.18	(-0.34, -0.02)	**0.026**	-0.15	(-0.31, +0.00)	0.051

Supplementary Table 3. Multivariable linear regression models evaluating the effect of anti-hypertensive medications on TBS and on BMD T-scores at lumbar spine, total hip and femoral neck, restricted to hypertensive patients not currently treated with anti-osteoporotic drugs (N = 3421). The excluded patients were 165 (136 on bisphosphonates, 28 on raloxifene, 1 on teriparatide, none on other treatments). Significant p-values are highlighted in bold. Abbreviations: ACE, angiotensin-converting enzyme; ARBs, angiotensin II receptor blockers; CCBs, calcium channel blockers; CI, confidence interval; MRAs, mineralocorticoid receptor antagonists; PPIs, proton pump inhibitors; β-coeff, β-coefficient.

Parameter	Lumbar spine TBS			Lumbar spine T-score			Total hip T-score[a]			Femoral neck T-score[a]		
	**β-coeff**	**95%CI**	**p-value**	**β-coeff**	**95%CI**	**p-value**	**β-coeff**	**95%CI**	**p-value**	**β-coeff**	**95%CI**	**p-value**
ACE-inhibitors	+0.000	(-0.011, +0.012)	0.933	+0.11	(-0.06, +0.27)	0.190	+0.12	(-0.02, +0.27)	0.097	+0.10	(-0.04, +0.24)	0.169
ARBs	+0.005	(-0.010, +0.019)	0.511	+0.27	(+0.09, +0.45)	**0.004**	+0.23	(+0.03, +0.43)	**0.024**	+0.21	(+0.05, +0.37)	**0.014**
Dihydropyridine CCBs	-0.011	(-0.025, +0.004)	0.147	-0.08	(-0.24, +0.08)	0.302	-0.14	(-0.27, -0.02)	**0.026**	-0.17	(-0.26, -0.08)	**<0.001**
Non-dihydropyridine CCBs	-0.022	(-0.046, +0.002)	0.076	-0.12	(-0.34, +0.11)	0.294	-0.14	(-0.40, +0.12)	0.269	-0.04	(-0.20, +0.13)	0.652
Beta blockers	+0.001	(-0.011, +0.013)	0.883	+0.08	(-0.09, +0.24)	0.342	+0.05	(-0.07, +0.18)	0.397	+0.02	(-0.08, +0.12)	0.687
Alpha blockers	+0.020	(-0.009, +0.049)	0.165	+0.19	(-0.20, +0.58)	0.325	-0.07	(-0.39, +0.26)	0.683	-0.11	(-0.40, +0.18)	0.460
Loop diuretics	-0.030	(-0.075, +0.016)	0.193	+0.04	(-0.41, +0.50)	0.846	-0.35	(-0.63, -0.07)	**0.016**	-0.16	(-0.39, +0.07)	0.167
Thiazide(-like) diuretics	+0.009	(-0.005, +0.024)	0.191	+0.30	(+0.12, +0.48)	**0.002**	+0.04	(-0.11, +0.20)	0.551	+0.04	(-0.08, +0.16)	0.465
MRAs	+0.017	(-0.030, +0.064)	0.456	+0.22	(-0.37, +0.80)	0.456	-0.02	(-0.61, +0.56)	0.935	-0.05	(-0.52, +0.41)	0.817
Other potassium-sparing diuretics	-0.004	(-0.037, +0.029)	0.823	-0.03	(-0.35, +0.29)	0.862	+0.11	(-0.12, +0.35)	0.332	+0.07	(-0.12, +0.27)	0.456
Alpha-2 agonists	-0.041	(-0.131, +0.049)	0.358	-0.09	(-0.95, +0.77)	0.826	+0.04	(-0.62, +0.70)	0.909	-0.14	(-0.68, +0.40)	0.600
Direct vasodilators	+0.020	(-0.061, +0.102)	0.616	-0.07	(-0.93, +0.80)	0.877	+0.12	(-0.59, +0.84)	0.726	+0.33	(-0.46, +1.13)	0.394

[a] Missing outcome data in 196 (5.7%) subjects

Notes: Medications are modeled as binary variables. All analyses are adjusted for age, sex, menopausal status, race/ethnicity, income, habitual physical activity, smoking status, BMI category, diabetes mellitus, eGFR category, history of liver disease, recent hospitalization (≤ 1 year), dietary calcium intake, 25OH-VitD levels, PPI treatment, hormone-blocking treatment, and history of chronic GC treatment.
